# Therapeutic effects of hydro-alcoholic extract of *Achillea wilhelmsii C. Koch* on indomethacin-induced gastric ulcer in rats: a proteomic and metabolomic approach

**DOI:** 10.1186/s12906-019-2623-4

**Published:** 2019-08-07

**Authors:** Mehdi Koushki, Reyhaneh Farrokhi Yekta, Nasrin Amiri-Dashatan, Masoumeh Dadpay, Fatemeh Goshadrou

**Affiliations:** 1 Department of Clinical Biochemistry, Faculty of Medicine, Tehran University of Medical Sciences, Tehran, Iran; 2 Proteomics Research Center, Shahid Beheshti University of Medical Sciences, Tehran, Iran; 3 Department of Pathology, AJA University of Medical Sciences, Tehran, Iran; 4 Department of Basic Sciences, Faculty of Paramedical Sciences, Shahid Beheshti University of Medical Sciences, Tehran, Iran

**Keywords:** *Achillea wilhelmsii*, Gastric lesion, Indomethacin, Metabolomics, Proteomics

## Abstract

**Background:**

Gastric ulcer is one of the most prevalent diseases worldwide. In Iranian folk medicine, *Achillea wilhelmsii* (AW) is used as a treatment for gastric ulcer. Previous reports also mentioned Antiulcerogenic properties for this herbal plant. This study investigated the therapeutic effects of *Achillea wilhelmsii C. Koch* extract on indomethacin-induced gastric lesion in rats, from both proteomic and metabolomic perspectives.

**Methods:**

The rats were divided into 4 groups. Gastric ulceration was induced by a single dose of indomethacin (45 mg/kg) by oral gavage. An amount of 800 mg/kg of AW extract was administered orally. Serum and tissue samples were collected for further investigations. The metabolomic study was performed by ^1^H-NMR CPMG spectrometry. Proteomic analysis was also executed by using two dimensional gel electrophoresis (2DE) followed by liquid chromatography coupled to tandem mass spectrometry (LC-ESI/MS/MS). Real time PCR was used to confirm some of the genes.

**Results:**

The macroscopic and microscopic investigations confirmed the effectiveness of the AW extract. There was a panel of metabolites which showed alteration during gastric lesion development. The levels of some of these metabolite reversed nearly to their control values after the administration of AW extract. There were also changes in the levels of some proteins including Alb, Fabp5, Hspb1, Tagln, Lgals7, Csta and Myl9 which were reversed after AW administration.

**Conclusions:**

Our findings suggested that *Achillea wilhelmsii C. Koch* extract could be a potential therapy to be used for indomethacin-induced gastric lesion treatment in the future. However, further investigations are needed to confirm the results.

**Electronic supplementary material:**

The online version of this article (10.1186/s12906-019-2623-4) contains supplementary material, which is available to authorized users.

## Background

Gastric ulcer is a benign lesion on the mucosal epithelium which is considered the most prevalent gastrointestinal disorder affecting 5% of the population worldwide. However, its management has turned into a major challenge in recent years [1, 2]. According to previous reports, dietary habits, smoking, non-steroidal anti-inflammatory drugs (NSAIDs), psychological stress and *Helicobacter pylori* infection are the most important causes of developing peptic ulcers [3]. NSAIDs are extensively used as anti-inflammatory drugs, however, their administration corresponds to 25% of gastric ulcer cases [4]. Indomethacin as one of the main NSAIDs induces gastric ulcer via inhibition of Prostaglandin E2 (PGE2) synthesis, inhibiting the release of cyclooxygenase-1, free radical production, decreasing gastric nitric oxide level and apoptosis induction in gastric cells [5]. Indomethacin became the first choice for the creation of an experimental gastric ulcer model because of its higher ulcerogenic potential than other NSAIDs [6]. Currently, there are several antiulcer drugs such as ranitidine and omeprazole that are used to manage NSAIDs-induced gastric ulcers. However, these therapies have major side effects and the search for non-toxic and easily accessible drugs is still in progress [7]. In recent years, the use of herbal medicine has attracted interest among the researchers for the treatment of different diseases. Plant extracts are potential sources of new therapeutic agents and several drugs have also been introduced for the treatment of gastrointestinal disorders [8, 9]. *Achillea*, a genus belonging to the *Asteraceae* family, embraces more than 100 species worldwide [10]. *Achillea wilhelmsii* (AW) widely grows in various parts of Iran, especially in the central and western areas [11]. This herb is a rich source of flavonoids and sesquiterpene lactones. It is also an important plant in traditional Persian medicine. Various compounds were isolated from this plant including alkaloids, volatile oils, saponin, tannins, resin, sterols and carbohydrates [11]. *Achillea wilhelmsii C. Koch* has previously shown to have anti-hyperlipidemic, anti-hypertensive and anti-mycobacterial properties. Rashidi et al. reported anti-inflammatory and healing effects of *Achillea* in the treatment of indomethacin-induced gastric ulcer in rats [12]. The study by Cavalcanti suggested an anti-ulcer effect for this plant [13]. Potrich et al. showed that using 30, 100 and 300 mg/kg *Achillea millefolium* could inhibit ethanol-induced gastric lesions and could also significantly promote the regeneration of the gastric mucosa after ulcer induction [14]. Muscarinic receptors located on the parietal cells which mediate acid secretion, are of the muscarinic M3 subtype [15]. A Brazilian study has reported that stimulation of muscarinic receptors (M3) of parietal cells rises the levels of gastric peptides which leads to histamine secretion and reduction of blood flow to the stomach mucosal layer. It would in turn, increase gastric secretion and reduce the protective factors of the stomach. Furthermore, the authors of that study suggested that the hydro-alcoholic extract of this species reduces the volume and acidity of the gastric juice via blockage of the main receptors presented in the parietal cell [16]. Recently, “omics” tools including proteomics and metabolomics along with bioinformatics analysis have expanded insights into comprehending the molecular mechanisms of diseases, biomarker discovery and understanding drug effects in disease treatment [17] which could, therefore, help to find mechanisms of new herbal drugs in gastrointestinal disorders. Hence, the aim of this study was to appraise the effects of the hydro-alcoholic extract of *Achillea wilhelmsii C. Koch* on proteomic and metabolomic profiles of indomethacin-induced gastric ulcer in rats.

## Methods

**Fig. 1:**
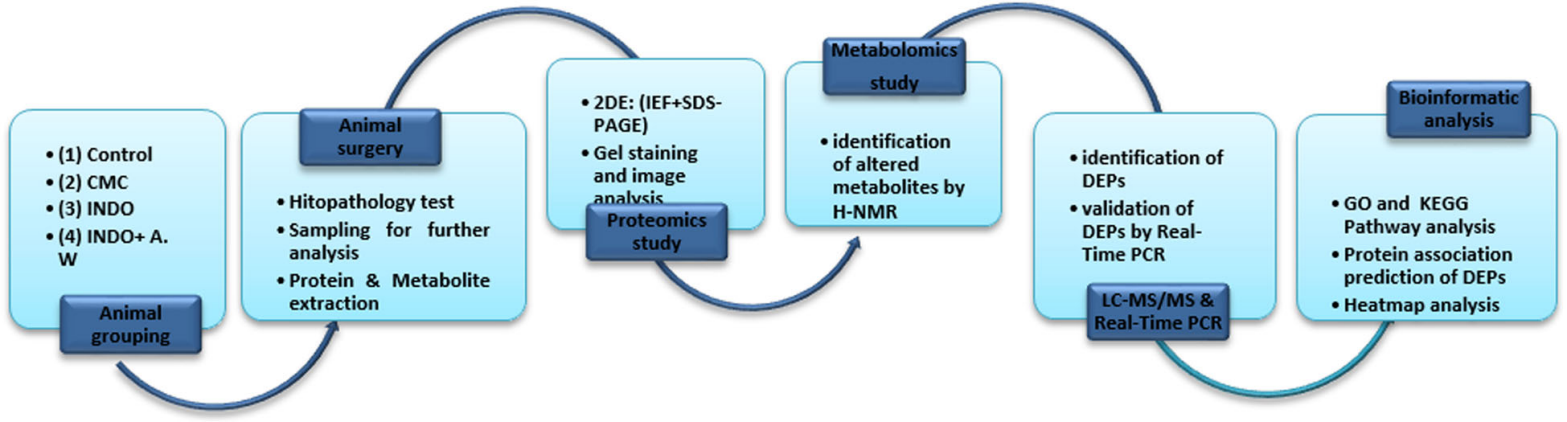
The workflow of the metabolomic and proteomic analysis of the therapeutic effects of *A. Wilhelmsii* extract on indomethacin-induced gastric ulcer. CMC: carboxymethyl cellulose; INDO: indomethacin; A.W: *Achillea Wilhelmsii*; ^1^H-NMR: nuclear magnetic resonance spectrometry; 2DE: 2-dimensional electrophoresis; IEF: isoelectric focusing; SDS-PAGE: sodium dodecyl sulfate; DEPs: differential expressed proteins; GO: gene ontology

An outline of the workflow of this study is summarized in Fig. 1.

### Chemicals and drugs

The Indomethacin used in this study was purchased from Sigma-Aldrich Co. (St. Louis, MO, USA) which was suspended in carboxy-methyl cellulose (CMC) for oral gavage. Criterion precast polyacrylamide gels, TGS and XT MES electrophoresis running buffers, Ready Strip™ IPG strips, mineral oil, dithiothreitol (DTT), iodoacetamide (IA), CHAPS, Biolytes and urea were purchased from Bio-RAD (Hercules, CA, USA).

### Plant extract: hydro-alcoholic extraction

*Achillea wilhelmsii C. Koch* plant was obtained from Neyshabour city in Khorasan Razavi province and was identified by Herbarium of Shahid Beheshti University (voucher No. 142–2019-4). The Dried aerial parts of the plant (300 g) were soaked in 50% ethanol for 24 h and the solution was filtered using paper filters. The resulting solution was dried using a 40 °C oven for 36 h. A single dose of 800 mg/kg was prepared by dissolving the dried extract in distilled water. We selected the 800 mg/kg dose of *Achillea wilhelmsii C. Koch* extract based on the findings of our previous studies on *Achillea wilhelmsii C. Koch* [18].

### Experimental design

The subjects involved in this study were 20 male wistar rats, weighing between 180 and 220 g which were randomly assigned to four groups consisting of 5 rats each. They were housed 3 to 4 per cage with 12-h light/dark cycles at 24 °C temperature. During this period, animals had free access to food and water. This study was conducted in accordance with the policies stipulated in the Guide for the Care and Use of Laboratory Animals (NIH). The present study was also approved by the clinical ethics committee of the faculty of paramedical sciences of Shahid Beheshti University of Medical Sciences (with Ethical code: IR.SBMU.RETECH.REC.1395.885). The rats were allowed 1 week of acclimatization to their environment. Subsequently, the animals were grouped as follows: Group 1: Normal controls receiving deionized water (1 ml), Group 2: Normal controls receiving 1% CMC solution, Group 3: rats receiving 45 mg/kg body weight of indomethacin (I), Group 4: rats receiving 45 mg/kg body weight of indomethacin + 800 mg/kg of body weight of AW extract (I + AW).

All the rats were treated for a total of 10 days. The animals were fasted for 24 h earlier. Then, Six hours after the administration of indomethacin, the Blood samples were collected from orbital sinus by a capillary tube and all rats were euthanized by administering intraperitonal ketamine and xylazine at doses of 60 and 20 mg/kg respectively and sacrificed; their stomachs were dissected and rinsed with normal saline. After evaluating the stomach ulcers macroscopically, Histopathological examinations was also performed on the stomach tissue.

### Lesion induction in animals and lesion index measurement

Gastric lesion was induced in the animals according to the following procedure; the rats were denied access to food 24 h prior to ulcer induction, but water was freely available. Then, they were administered a single oral dose of indomethacin (45 mg/kg body weight). Ulcers were manifested 6 h after indomethacin administration. The stomachs were opened along the greater curvature and rinsed with water to remove gastric contents and blood clots and examined to assess the formation of ulcers. The number of ulcers in each stomach was counted and averaged to calculate the ulcer index number according to the following formulae 1: Ulcer Index = (U/N) × 100, where U is the number of ulcers in the stomachs of each group rats and N is the number of rats in this group [19, 20].

### Histopathological examination

After rats’ surgery, gastric tissue samples of 3 rats/group were randomly selected for histopathologic studies. Tissues were completely washed with normal saline to remove any contaminants and were then fixed in buffered 10% formalin and processed for histopathological examination. Briefly, five micrometer-thick paraffin sections were prepared and stained with hematoxylin and eosin (H&E) for microscopic assessment by blind pathologist.

### Sample preparation for metabolomic study

For preparation of the stomach tissue extracts, 300 mg of the frozen tissues were grounded completely in liquid nitrogen and homogenized in 1 ml of 2:1 v/v Methanol/Chloroform solution. After that, 1 ml of 1:1 v/v Chloroform/H_2_O was added and the solution was centrifuged for 20 min at 15,000 g and 4 °C. 600 μl of the upper phase was then collected and lyophilized. For NMR analysis, the lyophilized tissue extract was dissolved in 600 μL of phosphate buffer solution containing 80% D_2_O, 2% TSP (trimethylsilylpropionate), 4% KH_2_PO_4_ and 0.01% NaN_3_. Serum samples were also dissolved in 400 μl of this buffer for NMR analysis.

### ^1^H-NMR spectrometry

The one-dimensional proton nuclear magnetic resonance spectrometry (^1^H-NMR) analysis was performed on a Bruker Avance 400 MHz instrument equipped with 5 mm probe at 298 K. The Carr-parcel-Meiboomian-Gill (CPMG) platform was used by a standard pulse sequence irradiating residual water peak, relaxation delay of 2 s and total T2 relaxation time of 60 ms. Other features of the spectrum collection included 150 total scans, spectral width of 8389.26 Hz, 90° pulse width and 0.5 Hz line broadening prior to Fourier transformation. The spectra were phased, base-line corrected and were referenced to the peak of TSP as the standard at 0 ppm. The NMR spectra were binned in the range of 0.3 and 9.5 as 0.01 ppm parts and were normalized and log-transformed. The region between 4.5 and 5.5 ppm was also omitted for water signal suppression. The NMR spectra were deconvoluted by ProMetab software in MATLAB (version 8.1.0.604, Mathworks, Cambridge, UK).

### Statistical analysis for metabolomic study

The data matrix resulted from ^1^H-NMR spectrometry was used to perform multivariate statistical analysis to identify the most significant relevant metabolites differentiating normal controls, I and I + AW groups. Multivariate statistical analysis included principal component analysis (PCA) and partial least square-discriminant analysis (PLS-DA) which were used to find significantly altered metabolites and also to build a predictive model. The PLS-DA modeling was executed by the “Tool for statistical analysis on Microsoft Excel”, a macros written by Visual Basic which is freely available at (http://prime.psc.riken.jp/menta.cgi/prime/index). The PCA analysis was performed in R (v.3.3.2).

### Metabolite identification

The variables resulted from the PLS-DA model which had *p*-values less than 0.05 and VIP values more than 1 were considered significant. The metabolites were then identified using relevant databases of NMR metabolomics including BMRB [21] and HMDB [22]. The tolerance for searching the spectral bins was ±0.01 ppm.

### Sample preparation for two-dimensional gel electrophoresis (2DE)

Fresh gastric tissues were snap frozen in liquid nitrogen and stored at − 80 °C until use.Tissue samples were washed twice by PBS and 10% Protease Inhibitor and then homogenized bypestle in lysis buffer containing 7 M Urea, 2 M Thiourea, 4% CHAPS (3-(3-Cholamidopropyl) dimethylammonio)-1-propanesulfonic acid), 40 mM Tris, 50 mM DTT (Dithiothreitol) and Protease Inhibitor (one tablet in 2 ml lysis buffer). Lysates were centrifuged at 15000×g for 15 min at 4 °C. Protein concentrations were determined by Bradford assay. 1200 μg from each sample were resuspended in rehydration buffer containing 8 M urea, 4% CHAPS, 0.2% Ampholyte, 50 mM DTT for 16 h. The 7 cm immobilized nonlinear gradient strips (pH = 3–10) (Bio-Rad, Hercules, CA, USA) were used in this study for the first dimension of 2DE (IEF). After IEF, proteins were submitted immediately to the second dimension separation in 12% acrylamide gels (1.5 mm thick) utilizing BioRad system. In order to improve the protein transfer from the first to second dimension, focused strips were equilibrated for 15 min in equilibration solution (containing 20% glycerol, 2% SDS, 6 M urea, 50 mM Tris-HCl and 2% DTT) and subsequently in the same buffer containing 2.5% iodoacetamide instead of DTT for 15 min. Electrophoresis was performed initially for 30 min at 16 mA/gel and then 6 h at 24 mA/gel.

### Gel staining and image analysis

Protein spots were stained by Colloidal Coomassie Brilliant Blue (CBB, G-250, Bio-Rad, USA). The stained gels were scanned and analyzed by the same spot prognosis software for identification of differentially expressed proteins in different study groups. Statistical analysis of protein variation was carried out using ANOVA test and significant expressions (fold-change> 2 and *p*-value< 0.05) were identified.

### LC-MS/MS analysis

Differentially expressed protein spots between “C & I” and “I & I+AW” groups were detected, excised manually from the stained gels and analyzed by LC-MS/MS. Gel spots were dehydrated in 50 mM Tris, pH 8.0, 50% acetonitrile and rehydrated with 10 mM DTT for 15 min at 65 °C. Proteins were then alkylated with 15 mM idoacetamide for 30 min in the dark and at room temperature. The spots were dehydrated again to remove excess reagents and were rehydrated in 50 mM Tris, pH 8.0 and 1 μg of Trypsin/LysC. The digestion was carried out overnight at 37 °C with agitation. Peptides were extracted from the gel by twice dehydration with 50% acetonitrile in 5% formic acid for 30 min. Acetonitrile was evaporated by speed-vac and the remaining peptides were purified by reversed phase solid phase extraction before LC-MS/MS analysis. Acquisition was performed with a ABSciex TripleTOF 5600 (ABSciex, Foster City, CA, USA) equipped with an electrospray interface with a 25 μm i.d. capillary and coupled to an Eksigent μHPLC (Eksigent, Redwood City, CA, USA). MS spectra were searched using the Mascot search engine (Matrix Science Inc., Boston, MA, USA). The International Protein Index (IPI) rat database (http://www.ebi.ac.uk/International_Protein_Index) was also used for peptide and protein identification. General protein identification was based on two or more peptides whose ion scores surpassed the statistical threshold (*p* < 0.05).

### Quantitative real-time RT-PCR verification

In order to verify the differentially expressed proteins obtained from the proteomic analysis, four proteins, including Galectin-7, heat shock protein beta-1, transgelin and cystatin-A, were selected randomly and their relative expressions were evaluated by quantitative real-time PCR. Total RNA was extracted from tissue samples using RiboEx reagent (GeneAll Biotech, Korea) according to the manufacturer’s instructions. The quantity of RNA was evaluated using Nano-drop (ND-1000, Thermo Scientific Fisher, US). Complementary DNA (cDNA) was synthesized from 3 μg of total RNA using RevertAid™ First Strand cDNA Synthesis Kit (Thermo Fisher Scientific Inc) following the manufacturer’s instructions. Real-time-PCR was conducted to examine the relative differential expression of genes between the examined groups. Target genes primers were designed by Gene Runner software version 6.5.50 (http://www.generunner.net). The sequences of primers used in Real-Time PCR are presented in Table 1. β-Actin gene was selected for normalization purposes and referred to as internal control. RT-PCR was performed in 20 μl reactions containing 1 μl cDNA target, 100 nM forward and reverse primers and 1x SYBR Green RealQ Plus Master Mix (Ampliqon, DK-5230 Odense M, Denmark). Experiments were carried out in duplicate using a StepOne TM RealTime PCR System (Applied Biosystems, Life Technologies, USA). The PCR conditions were as follows: activation at 95 °C for 10 min, amplification at 95 °C for 15 s, 60 °C for 1 min for 40 cycles. The relative values of the expression levels of each gene were determined based on the threshold cycle (CT) value of the target gene, normalized to that of reference gene (β-Actin) using the 2^-∆∆CT^ equation and the level of acceptable significance was 95% (*P* < 0.05).

**Table 1:** Sequence of primers, which were used for Real-Time RT-PCR

Gene name	Primer sequence
Galectin-7	F: TAAACCTGCTATGCGGCGAG R: TGCCTTGCTGTTTGGTGTTG
Heat shock protein beta-1	F: ATCACTGGCAAGCACGAAGA R: GAGCGTGTATTTCCGGGTGA
Transgelin	F: ATCCTATGGCATGAGCCGTG R: CAGGCTGTTCACCAACTTGC
Cystatin-A	F: TGAAAAATTCGAAGCCGTTGAGT R: CATGTCCCACATCCATCTTAATG
β-Actin	F: ACGAGGCCCAGAGCAAGAG R: GGTGTGGTGCCAGATCTTCTC

*F* forward, *R* reverse

## Results

### Morphological and histopathological examinations

**Fig. 2:**
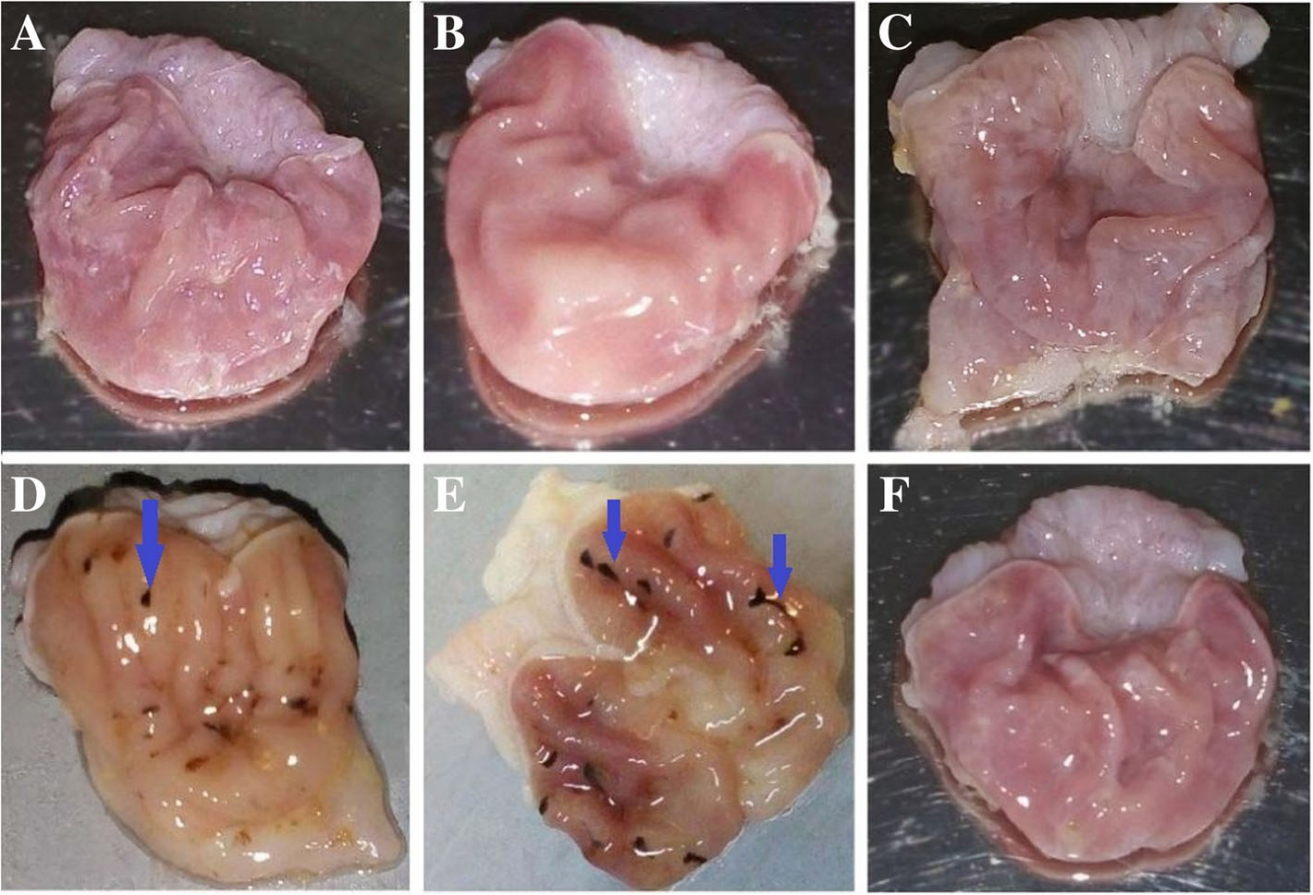
The macroscopic images of the stomach from normal control rats (**a**, **b**), normal controls receiving CMC (**c**), indomethacin-induced gastric ulcer (**d**, **e**) and ulcerated rats receiving AW extract (**f**). The rats showed normal mucosa after treatment with AW extract. Arrows show linear and focal haemorrhagic areas

**Fig. 3:**
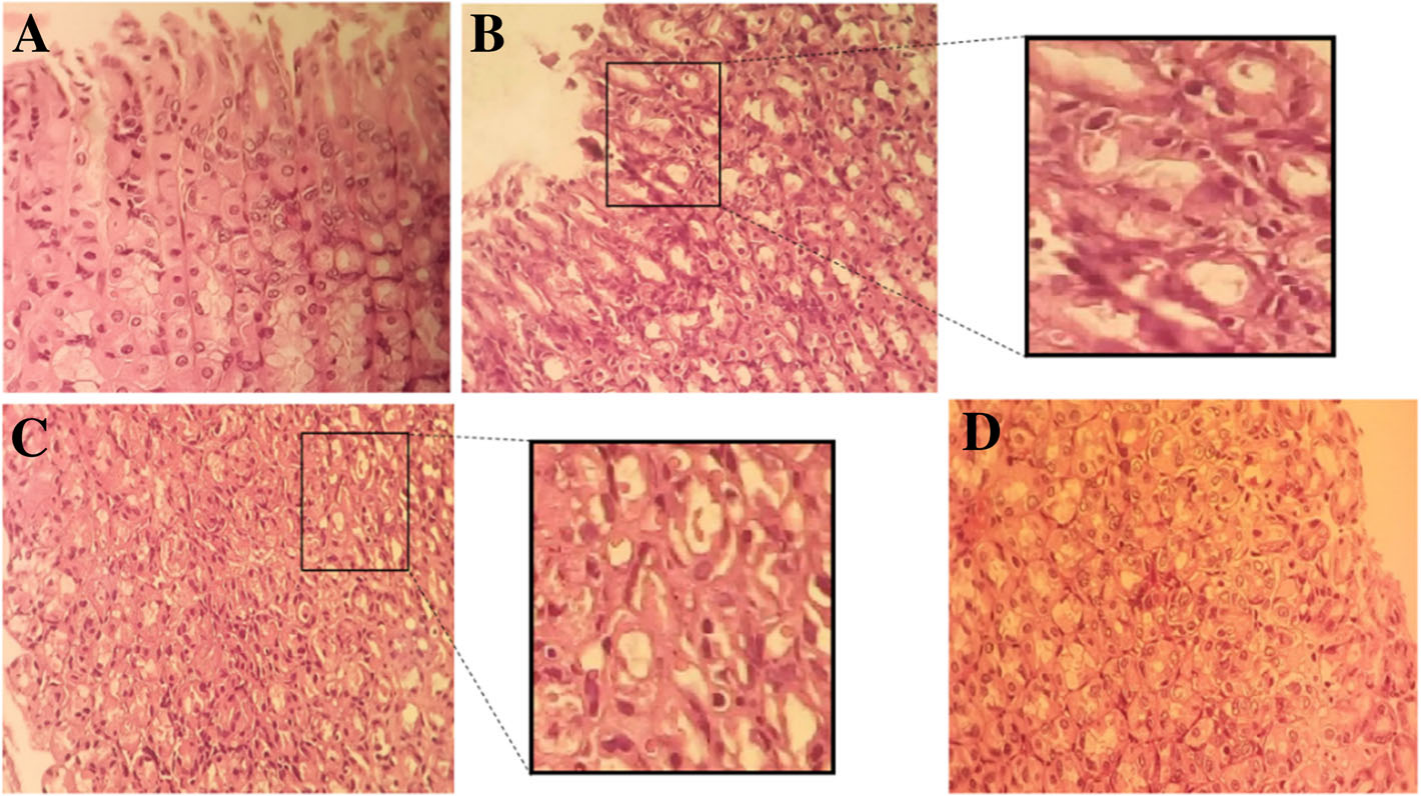
The gastric mucosa appearance stained with H&E (100x magnified) in (**a**) normal, (**b**, C) indomethacin-induced lesions and (**d**) gastric ulcer after treatment with AW extract. Normal sections have intact epithelium with distinct chief and parietal cells where ulcer areas show epithelium damage and infiltration of leukocytes

Figure 2 shows the macroscopic appearance of the stomachs from normal rats (part A, B), rats receiving CMC as the indomethacin vehicle (part C), normal rats after the administration of indomethacin (45 mg/Kg) (part D, E) and indomethacin-treated stomachs after receiving AW extract (part F). As can be seen, indomethacin-treated rats indicate hemorrhagic areas, where AW-treated rats show normal mucosa. The lesion Index which was measured for the ulcer rats was 2500 according to the Formulae 1 where this value was 0 for the other groups. The histological micrographs of the rat stomachs are also presented in Fig. 3. The tissue sections were stained with hematoxylin and Eosin (H&E) with 100× magnification. Normal control rats showed intact and normal gastric mucosa where the indomethacin-treated rats showed erosion of the gastric mucosa superficial layer and infiltration of the leukocytes. The lesion areas appeared normal after treatment with AW extract.

### Comparative analysis of proteome changes

**Fig. 4:**
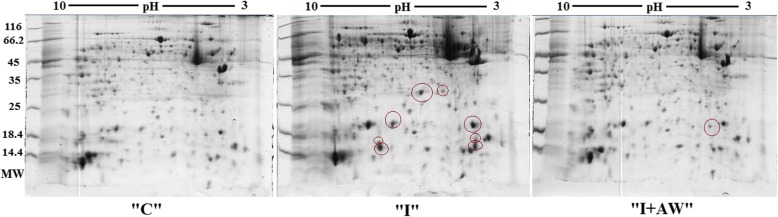
The proteome profiles obtained from 2DE analysis of stomach tissues for normal (“C”), rats with indomethacin-induced ulcer (“I”) and ulcerated rats after treatment with AW extract (“I + AW”). The differentially expressed proteins are denoted by circles

**Fig. 5:**
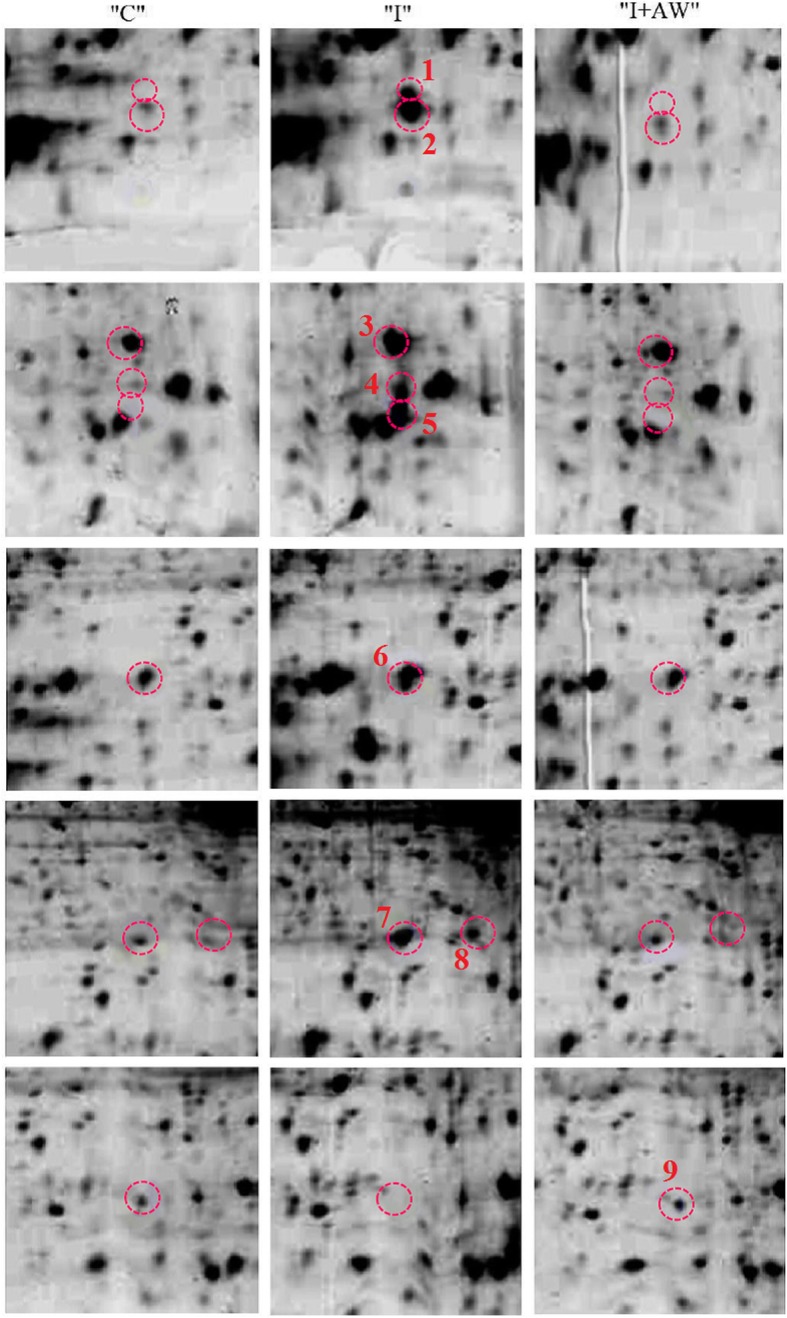
Enlarged differentially expressed spot images from the respective gels. The proteins (No. 1–9) are as follows: 1. Lgals7, 2. Fabp5, 3. Myl9, 4, 5. Csta, 6. Tagln, 7, 8. Hspb1, 9. Alb. (“C”: control group, “I”: rats with indomethacin-induced gastric ulcer, “I + AW”: ulcerated rats treated with AW extract)

To explore the molecular mechanisms underlying the therapeutic effects of hydro-alcoholic extract of *Achillea wilhelmsii C. Koch* on indomethacin-induced gastric ulcer in rat models, 2DE-based proteomic approach was used. The representative images of the 2DE gels are provided in Fig. 4. The significant differentially expressed proteins between groups were then identified by LC-ESI-MS/MS. These proteins included serum Albumin (Alb), Fatty acid binding protein/epidermal (Fabp5), Heat shock protein beta-1 (Hspb1), Transgelin (Tagln), Galectin-7 (Lgals7), Cystatin-A (Csta) and Myosin regulatory light polypeptide 9 (Myl9). The images of protein spots are presented in Fig. 5. Detailed properties of the identified proteins are also shown in Table 2.

**Table 2:** List of the statistically significant (fold change> 2, P < 0.05) gastric tissue proteome changes in C, I and I + AW obtained from 2-DE gels after identification by LC-MS/MS analysis

Uniprot ID	Protein	Gene symbol	Sequence Coverage (%)	Peptides (95%)	Fold change (I/C);(I + AW/I)
P02770	Serum albumin	Alb	12.17	5	0.25; 3.88
P55053	Fatty acid binding protein 5	Fabp5	94.81	62	6.1; 0.18
P42930	Heat shock protein beta-1	Hspb1	90.3	71	4.43; 0.38
P31232	Transgelin	Tagln	81.09	92	2; 0.55
P97590	Galectin-7	Lgals7	80.15	15	4; 0.26
P01039	Cystatin-A	Csta	85.4	39	4.7; 0.22
Q64122	Myosin regulatory light polypeptide 9	Myl9	71.35	79	2; 0.53

(“C”: control group, “I”: rats with indomethacin-induced gastric ulcer, “I + AW”: ulcerated rats treated with AW extract)

### Validation of the differentially expressed proteins by real-time RT-PCR

**Fig. 6:**
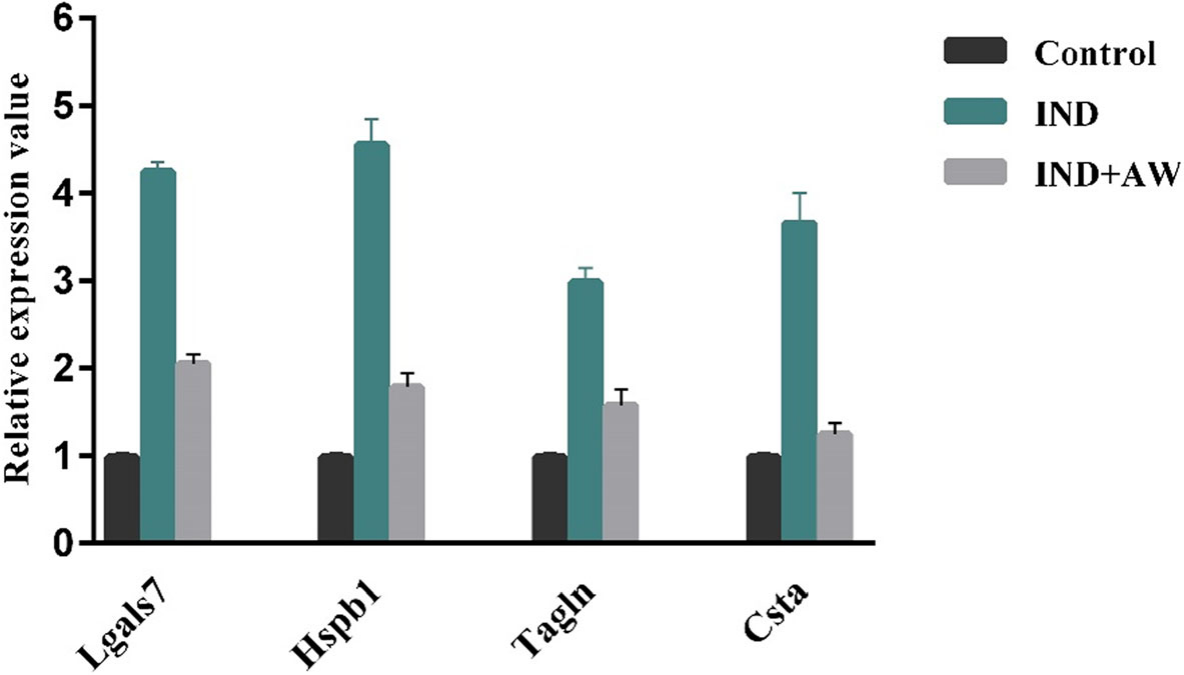
Relative gene expression patterns of 4 differentially expressed proteins between I, I + AW and control groups by Real-Time PCR. The expression of Actin was used as the internal control gene. The values are as mean ± SD of three independent experiments (*P* < 0.05). Lgals7: Galectin-7, Csta: Cystatin-A, Hspb1: Heat shock protein beta-1, Tagln: Transgelin. (“C”: control group, “I”: rats with indomethacin-induced gastric ulcer, “I + AW”: ulcerated rats treated with AW extract)

The transcript levels of some of the differentially expressed proteins including Galectin-1, Cystatin-A, Hspb-1 and Transgelin were confirmed using Real-Time RT-PCR (see Fig. 6).

### Comparative analysis of metabolic changes

**Fig. 7:**
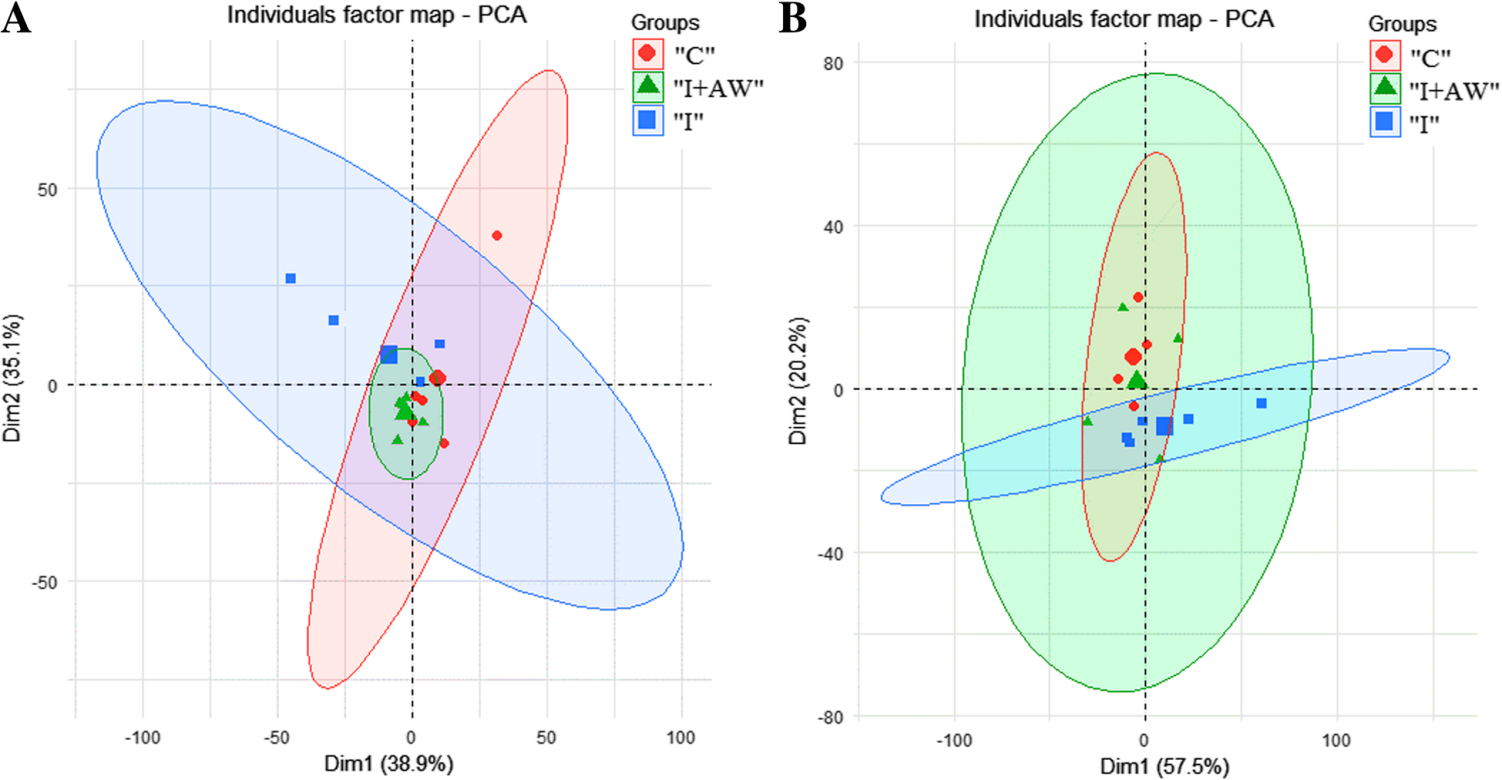
The PCA score plots discriminating (**a**) serum and (**b**) tissue samples comparing “C-I” and “I-I + AW” groups. The C and I + AW samples are clustered very close to each other which means the treatment was successful. (“C”: control group, “I”: rats with indomethacin-induced gastric ulcer, “I + AW”: ulcerated rats treated with AW extract)

**Fig. 8:**
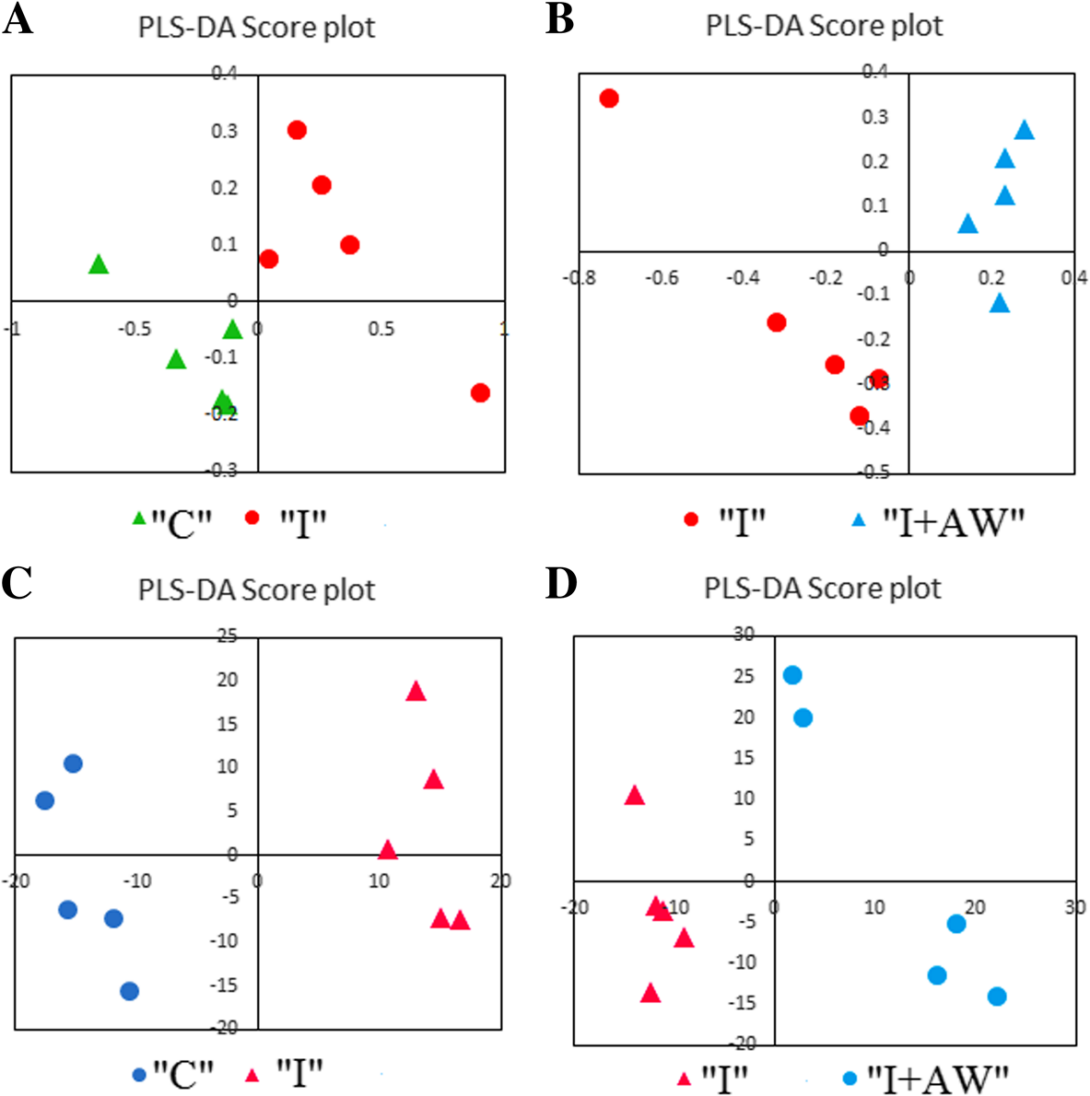
The PLS-DA score plots of serum (**a**, **b**) and tissue (**c**, **d**) samples for the comparison of “C-I” and “I-I + AW”. The models could successfully discriminate the study groups. (“C”: control group, “I”: rats with indomethacin-induced gastric ulcer, “I + AW”: ulcerated rats treated with AW extract)

**Fig. 9:**
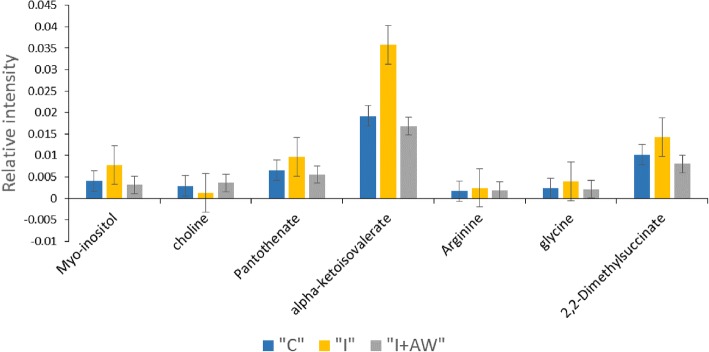
Levels of 7 serum metabolites reversed nearly to control values after treatment with AW extract. The Y-axis represents relative peak intensities of the NMR spectra. (“C”: control group, “I”: rats with indomethacin-induced gastric ulcer, “I + AW”: ulcerated rats treated with AW extract). Values are shown as mean ± SE

**Fig. 10:**
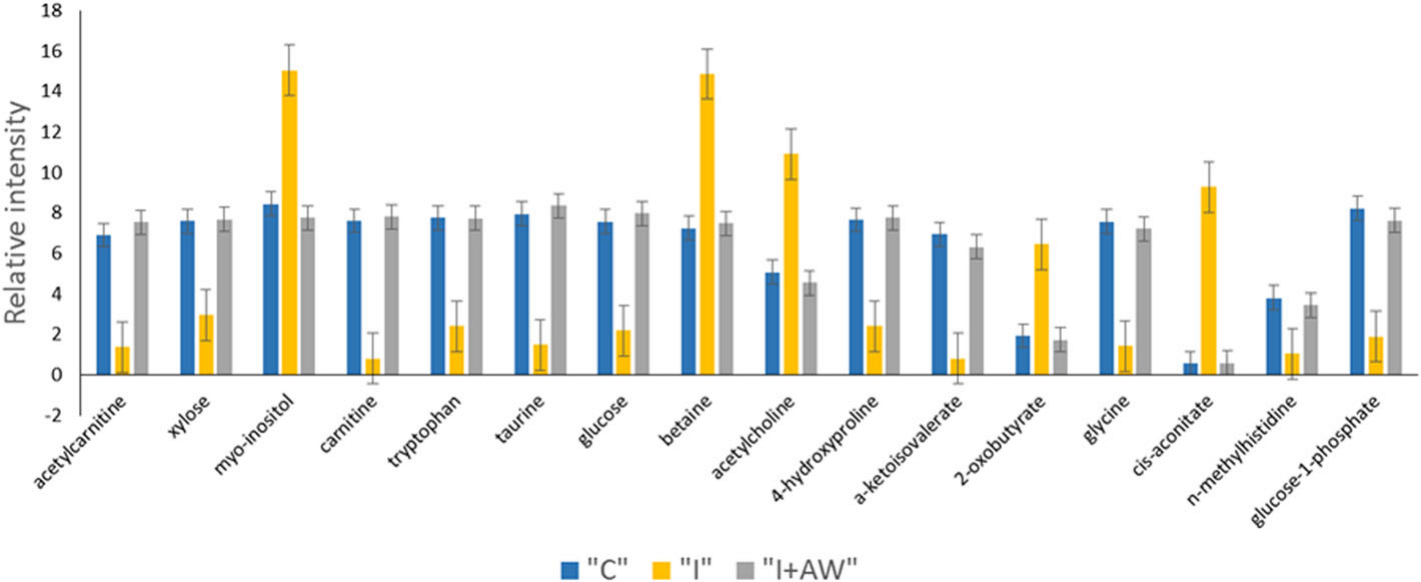
Stomach tissue levels of 16 metabolites reversed nearly to control values after treatment with AW extract. The Y-axis represents relative peak intensities of the NMR spectra. (“C”: control group, “I”: rats with indomethacin-induced gastric ulcer, “I + AW”: ulcerated rats treated with AW extract, Values are shown as mean ± SE.)

**Fig. 11:**
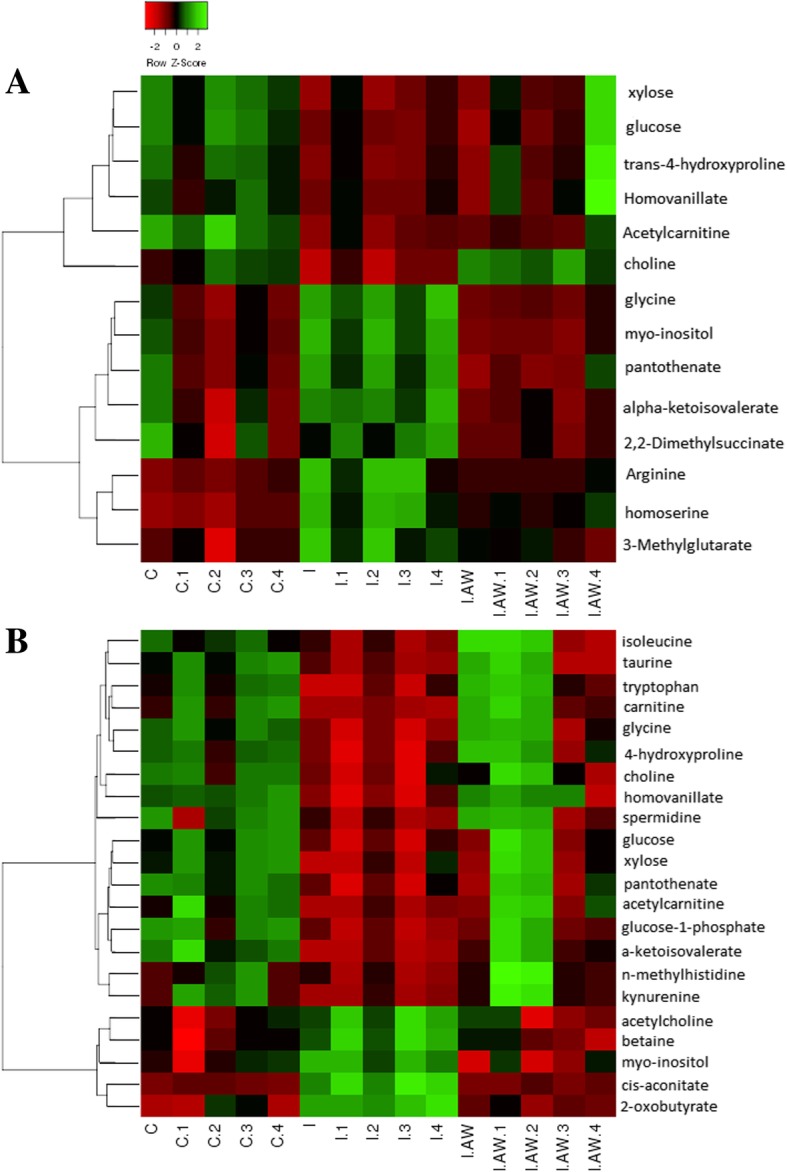
Heatmap clustering representation of differentially expressed metabolites between the study groups for serum (**a**) and tissue (**b**) samples. (“C”: control group, “I”: rats with indomethacin-induced gastric ulcer, “I.AW”: ulcerated rats treated with AW extract)

The therapeutic effects of *Achillea wilhelmsii* extract on indomethacin-induced gastric ulcer in rats were also studied from a metabolomic perspective. ^1^H-NMR spectrometry was used to find the most significantly altered metabolites in the groups of study. Multivariate statistical analysis was performed to find the significant relevant metabolite markers which mostly discriminated the groups. Principal component analysis (PCA) was the unsupervised method which determined the trends in the data and any possible outliers. PCA is used for dimension reduction of data matrix by making a linear combination of variables which are known as principal components. There were no significant alterations between controls and the rats receiving vehicle. Figure 7 shows the PCA results for the discrimination of C, I and I + AW groups. The first two principal components (PCs) for serum and tissue samples were as follows; a- For serum samples, the PC1 and PC2 were 38.9 and 35.1% respectively for the differentiation of the C, I and I + AW groups. b- For tissue samples, the PC1 and PC2 were 57.5 and 20.2% respectively for the differentiation of the C, I and I + AW groups. The first two components could differentiate the study groups for about near 80% which is acceptable. There were also no outliers in any of the groups and all the observations were within the 95% confidence interval ellipsoid. It was observed that in the PCA score plots in both serum and tissue samples, the I + AW and control groups were clustered very close to each other which means that the therapy could be successful. PLS-DA was also performed as the supervised analytical method to find the metabolites which caused significant variations between the study groups. The PLS-DA scatter score plots are shown in Fig. 8. The Q2_cum_ values (cumulative percent of variation in Y-matrix, predicted by the model) of the PLS-DA models were calculated. For serum samples, the Q2_cum_ was 0.6 for the differentiation of C and I, and 0.62 for the differentiation of I and I + AW. For the tissue samples, this value was 0.855 for the differentiation of C and I, and 0.917 for the differentiation of I and I + AW. The root mean square error (RMSE) values for the above comparisons were also 0.19, 0.179, 0.079 and 0.088 respectively. The Q2_cum_ and RMSE values demonstrated the model’s good predictive ability. The Operating Characteristic (ROC) curve and the “Y observed” versus “Y predicted” diagram for the PLS-DA models are provided in Additional file 1: Figure S1. The area under the curve (AUC) was 1 for both serum and tissue which showed the high accuracy of the PLS-DA models for the separation of the classes. The significant serum metabolites are shown in Table 3. The increasing metabolites in “I” compared with “C” included myo-inositol, pantothenate, α-ketoisovalerate, homoserine, arginine, 3-methylglutarate, glycine and 2,2-dimethylsuccinate. The decreased serum metabolites comparing “I” with “C” groups included glucose, xylose, choline, trans-4-hydroxyproline, acetylcarnitine and homovanillate. From the 14 serum metabolites which were altered in “I” group compared with “C”, 7 metabolites demonstrated a reverse expression trend after treatment with AW extract (Fig. 9). These included myo-inositol, choline, pantothenate, α-ketoisovalerate, arginine, glycine and 2,2-dimethylsuccinate. The significant tissue metabolites are shown in Table 4. For stomach tissue samples, the increased metabolites in group “I” compared with “C” included myo-inositol, betaine, acetylcholine, 2-oxobutyrate and cis-aconitate where decreased amounts of acetylcarnitine, xylose, carnitine, tryptophan, taurine, glucose, homovanillate, choline, pantothenate, isoleucine, 4-hydroxyproline, kynurenine, α-ketoisovalerate, glycine, spermidine, n-methylhistidine and glucose-1-phosphate were observed. From the above 22 metabolites which showed altered expression in tissue, 16 metabolites significantly altered after treatment with AW extract and showed reverse expression direction in “I + AW” group (Fig. 10). These 16 metabolites included acetycarnitine, xylose, myo-inositol, carnitine, tryptophan, taurine, glucose, betaine, acetylcholine, 4-hydroxyproline, α-ketoisovalerate, 2-oxobutyrate, glycine, cis-aconitate, n-methylhistidine and glucose-1-phosphate. Figure 11 shows the heatmap representation of the significantly altered metabolites between the study groups.

**Table 3:** Differentially expressed serum metabolites between normal controls and rats with gastric ulcer and between gastric ulcer and after treatment with AW extract

Metabolite	Matched chemical shifts	I vs. C		I + AW vs. I	
		VIP*	Fold (I/C)	VIP*	Fold (I + AW/I)
Glucose	3.405, 3.455, 3.735	2.294^b^	0.58	1.110	1.02
Xylose	3.425, 3.225	2.902^b^	0.54	1.510	1.08
Myo-inositol	3.525, 3.615	2.347^a^	1.93	3.725^c^	0.40
Choline	3.185, 3.515	1.542^a^	0.54	2.576^c^	2.20
Pantothenate	3.505	1.953^a^	1.65	3.355^a^	0.51
Trans-4-hydroxyproline	3.355, 3.475	2.036^a^	0.63	1.154	1.15
α-ketoisovalerate	1.135, 1.115, 2.985	4.836^a^	1.80	8.008^c^	0.46
Homoserine	2.085, 2.075, 3.745, 3.795	2.622^b^	1.61	1.725	0.82
Acetylcarnitine	3.625, 3.205	3.108^b^	0.40	1.341	1.12
Arginine	1.905, 3.785	1.296^b^	1.44	1.118^a^	0.75
3-Methylglutarate	0.905	1.046^a^	1.14	1.064	0.92
Homovanillate	3.445, 3.875	1.866^a^	0.80	1.440	1.18
Glycine	3.545	1.748^b^	1.72	2.420^c^	0.55
2,2-Dimethylsuccinate	1.155, 1.145	2.311^a^	1.47	5.332^b^	0.47

C: control group, I: rats with indomethacin-induced gastric ulcer, I + AW: ulcerated rats treated with AW extract. *a*: *p* < 0.05, *b*: *p* < 0.01, *c*: *p* < 0.001. * metabolites with VIP > 1 are considered

(*p < 0.05, **p < 0.01, ***p < 0.001)

**Table 4:** Differentially expressed tissue metabolites between normal controls and rats with gastric ulcer and between gastric ulcer and after treatment with AW extract

Metabolite	Matched chemical shifts	I vs. C		I + AW vs. I	
		VIP*	Fold (I/C)	VIP*	Fold (I + AW/I)
Acetylcarnitine	3.175, 3.595, 3.845, 3.615	3.009^b^	0.15	2.149^b^	7.61
Xylose	3.515, 3.215	2.677^a^	0.30	1.696^a^	3.09
Myo-inositol	3.265, 3.605	3.054^a^	1.49	5.288^a^	0.53
Carnitine	3.415	3.457^b^	0.05	2.486^b^	17.3
Tryptophan	3.295, 3.475	2.974^b^	0.26	1.953^a^	3.49
Taurine	3.405, 3.395	3.401^b^	0.13	2.276^b^	7.79
Glucose	3.505, 3.835	3.007^b^	0.24	1.945^a^	4.11
Homovanillate	3.455, 3.855	3.490^b^	0.26	1.873	4.09
Choline	3.525	2.232^a^	0.44	1.563	2.21
Pantothenate	3.425, 3.535	2.839^b^	0.27	1.805	3.87
Isoleucine	1.255, 3.665	2.360^b^	0.20	1.981	6.52
Betaine	3.255	3.546^a^	1.75	4.950^b^	0.48
Acetylcholine	3.205	3.070^a^	2.00	4.504^a^	0.37
4-hydroxyproline	3.375, 3.485, 3.495, 3.465	2.944^b^	0.26	1.880^a^	3.88
Kynurenine	3.705	2.424^a^	0.10	2.072	10.2
α-ketoisovalerate	1.135, 2.955	3.270^b^	0.05	2.296^a^	17.4
2-oxobutyrate	1.085, 1.065	2.755^b^	3.51	3.943^b^	0.25
Glycine	3.555	3.317^b^	0.17	2.140^a^	5.40
Spermidine	3.115, 3.165	3.002^a^	0.16	2.912	5.41
cis-aconitate	3.075, 3.085	4.307^c^	15.2	4.779^c^	0.05
n-methylhistidine	3.675	2.009^a^	0.17	2.040^a^	5.22
Glucose-1-phosphate	3.385	3.282^b^	0.17	2.186^a^	4.74

C: control group, I: rats with indomethacin-induced gastric ulcer, I + AW: ulcerated rats treated with AW extract. *a*: *p* < 0.05, *b*: *p* < 0.01, *c*: *p* < 0.001. *metabolites with VIP > 1 are considered

## Discussion

To the best of our knowledge, this is the first metabolomic and proteomic study to investigate the effects of *Achillea wilhelmsii* on indomethacin-induced gastric ulcer in rats. In the present study, gastroprotective effects of *Achillea wilhelmsii C. Koch* (AW) hydro-alcoholic extract were investigated on a group of rats ulcered with the vastly used non-steroid anti-inflammatory drug, indomethacin. Some previous studies confirmed the anti-oxidant and anti-ulcerative properties of *Achillea Wilhemsii* essential oil or extract [23, 24]. In a recent paper by Mohammadhosseini et al., a very complete and extensive overview on the biological activities and chemical composition of *Achillea* species including *Achillea Wilhemsii* was presented [25]. According to Khazneh et al., anti-inflammatory effects of AW hydro-alcoholic extract was related to some phenolic compounds such as isoschaftoside, schaftoside, vicenin-2, vicenin-3, caffeic acid, isoorientin, isovitexin, swertisin, 1β,10β-epoxydesacetoxymatricarin, leucodin, 5-demethylsinensetin and salvigenin [26]. According to Nekoei and Mohammadhosseini, the main components of AW aerial parts include 1,8-cineole, nerolidol, α-pinene, camphor, caryophyllene oxide, α-terpineol, linalool, camphene, *p*-cymene and limonene [27]. In this study, for the first time, we used two “omics” platforms including proteomics and metabolomics to seek the underlying mechanisms of the therapeutic effects of AW extract on indomethacin-induced gastric ulcer in rat models. Both macroscopic and histopathologic investigations confirmed the anti-ulcerogenic effects of the AW extract as was presented in Figs. 2 and 3. The metabolomics study showed a profile of significantly altered metabolites in the ulcer group which were reversed nearly to their control values after the administration of AW extract (as was shown in Figs. 8 and 9). From the altered metabolites in the rats treated with indomethacin, taurine level was significantly decreased. Taurine has important biological roles including antioxidant and anti-inflammatory function, membrane stabilization, modulation of intracellular free calcium concentration and protection against oxidant-mediated injury in several organs [28, 29]. Indeed, it protects against the drug-related gastric damage and colonic injury by its antioxidant properties. The results of several studies showed that taurine prevents gastric ulcer induced by indomethacin through lipid peroxidation inhibition and neutrophil activation [30] . Furthermore, taurine changes might imply the oxidative stress-related gastric ulceration. The results of our study showed decreased glucose levels in the indomethacin-treated group which might indicate that excessive glucose was consumed to ameliorate gastric injury. Cis-aconitate was a metabolite with increased amounts in I group compared with healthy controls. Aconitase, catalyses citrate to isocitrate via *cis*-aconitate in the tricarboxylic acid cycle. Because this compound is one of the intermediates in the TCA cycle, this alteration might suggest the disturbance of energy metabolism in GU [31]. In a previous study on gastric ulcer, alteration of cis-aconitate was observed which confirms this result [32]. Glycine was decreased in treated group tissues which could be due to elevated energy consuming in order to protect against gastric damage. Carnitine and acetylcarnitine were also decreased in indomethacin-induced gastric ulcer group. Acetyl-carnitine is broken down to carnitine which is used by the body to transport fatty acids into the mitochondria for breakdown [33]. According to numerous studies, carnitine and its derivatives are the main compounds in prevention of reactive oxygen formation and also have protective capacity in biological membranes against peroxidative stress. Free radicals and peroxidative stress are involved in gastric mucosal damage pathogenesis and based on previous data, carnitine contains beneficial effects by antiperoxidative function on ethanol-induced gastric mucosal damage [34]. It has been recently reported that carnitine has a gastroprotective effect on indomethacin-induced gastric mucosal injury in rats [35]. Spermidine was the other decreased metabolite in I group in this study. Spermidine content in mammalian cells has vital roles in the protection of cells from oxidative damages, cell proliferation, differentiation and apoptosis [36]. Given the very important role of spermidine in cell survival, its decreased levels in damaged stomach tissues seems logical. Previous investigations demonstrated inhibitory effects of polyamines such as spermidine on gastric ulceration and acid secretion [37]. In another study, the effect of polyamines on gastric ulceration in rats was also evaluated [38]. The other altered metabolite, pantothenic acid, is an essential nutrient that animals require in order to synthesize coenzyme-A, as well as to synthesize and metabolize proteins, carbohydrates and fats. Since pantothenic acid is the active part of coenzyme-A, it might play an important role in the metabolism of parietal cells. Pantothenic acid is one of the acetyl-CoA components, thus alteration of pantothenic acid level could affect acetyl-CoA metabolism and finally might influence the TCA cycle [39]. Methylglutarate was another metabolite with a slightly increased amount, which is shown to have a role in lipid metabolism pathways and lipid peroxidation. Its effects on gastric damage due to indomethacin consumption might be caused by producing free radicals. The other metabolite which was decreased in serum samples was hydroxyproline. Serum hydroxyproline content is an indicator of collagen metabolism or tissue degradation. Previous studies also confirmed that hydroxyproline is decreased during gastric ulceration induced by NSAIDs and showed the increased activity of the collagenase enzyme in gastric ulcer models [40]. 2-oxobutyrate was increased in the tissue samples after ulcer induction. 2-oxobutyrate is involved in some amino acids metabolism and might also enter the citric acid cycle by being converted to succinyl CoA. Its alteration might be caused by altered energy metabolism during ulceration. Decreased choline and increased acetylcholine contents were also observed in the metabolomic results. Acetylcholine inhibitors are proved to be a therapy against peptic ulcer as anti-secretory drugs [41] which confirms this increment of acetylcholine during ulcer induction.

The proteomic study results also showed that some proteins levels were reversed nearly to their control values after the administration of the AW extract. These proteins included Alb, Fabp5, Hspb1, Tagln, Lgals7, Csta and Myl9. Alb was decreased while the other proteins were increased after the induction of gastric ulcer. Serum albumin is shown to be a predictor of mortality in upper gastrointestinal bleeding. There were also some other studies which reported that hypoalbuminemia occurs during gastric bleeding [42]. The other protein with altered level was Hspb1. Heat shock proteins (HSPs) act as preventive factors during gastric ulcer formation and also in ulcer healing [43]. In a previous study, it was reported that induction of HSPs by a geranyl compound contributes to the gastro-protective activity of HSPs against NSAIDs [44]. The over-expression of Hspb1 might be a way to defend against aggressive factors such as free radicals or other stressors. Cystatin A (Csta) was the other increased protein in the ulcer group. Csta is a member of the cystatin family, mainly acting as cystein protease inhibitors. Another member of this family, cystatin C, was shown to be expressed in stomach neoplasms [45]. We observed increased levels of galectin-7 (Lgals7) in the lesion group. Over-expression of some galectins such as gal1 as an anti-inflammatory mediator was previously reported in gastric ulcer [46]. There were also alterations in Myl9 levels which is mainly a protein, enhancing tumor progression [47]. Increased Myl9 and Lgals7 levels might demonstrate a tendency in the ulcer areas to progress to cancerous lesions. Transgelin is another protein that was overexpressed in gastric ulcer group. The results of several studies on the tumorigenic and non-tumorigenic cell lines indicated that the metastatic potential of cancer stem cells arises from highly expressed transgelin. According to Lee et al., the expression level of transgelin was 25-fold higher in tumorigenic cells compared to non-tumorigenic cells [48]. In a comparative proteomics-based study on the gastric adenocarcinoma (GA) and paired non-neoplastic mucosa tissues, one of the upregulated proteins identified in GA was transgelin which was validated by immunohistochemistry and western blotting methods [49]. Furthermore, transgelin was introduced as a potential novel marker for gastric adenocarcinoma based on proteomics technology. Therefore, the up-regulation of transgelin in our results is in agreement with previous studies since gastric ulcer might be one of the principal risk factors of gastric cancer. The other significantly altered protein was the FABP5. FABP is a small intracellular protein that is increased in conditions such as inflammation and ischemia. Several types of FABP are described immunologically, including heart, intestinal, liver, epidermal, muscle and adipocyte FABPs. Intestinal FABP (I-FABP) is located exclusively in gastric epithelial cells and intestinal mucosa [50]. The correlation between overexpression of FABP5 and malignant potential of tumors and metastasis in several cancers such as prostate [51], esophageal [52] and breast cancer [53] has been previously reported. The up-regulation of this protein in indomethacin group could be due to high inflammation in the gastric mucosa of the indomethacin-treated group and infiltration of inflammatory cells. Oxidative stress and inflammatory mediators play a key role in gastric mucosal damage caused by NSAIDs and strong antioxidants can suppress oxidative damage related to NSAIDs [54]. Regarding the mechanism of the effect of inflammation and oxidant/anti-oxidant status in gastric damage, several studies have reported that the plant extract prevents gastric damages by attenuating inflammation and oxidant/antioxidant inequality [55]. Due to high phenolic contents of AW extract and their capacity to scavenge toxic radicals of oxygen, these compounds may be responsible for the AW antioxidant property [56]. Removing oxygen free radicals is one of the probable mechanisms underlying anti-inflammatory, anti-ulcerative and ulcer-healing properties of AW extract as increased metabolites such as taurine, carnitine and acetyl-carnitine (as important anti-inflammatory and antioxidant biomarkers in treatment group) confirm this conclusion.

## Conclusion

In summary, our study clearly demonstrated several functional categories of proteins and metabolites which might play important roles in the development of indomethacin-induced gastric ulcer and GU treatment. The results also extended our understanding of the GU treatment mechanisms by the AW extract. Our study suggested that the extract of *Achillea wilhelmsii* could be capable of healing the indomethacin-induced gastric ulcers probably due to anti-oxidative and anti-inflammatory mechanisms. In addition, the results illustrated the power of integrated proteomics and metabolomics approaches to reveal the biochemical and molecular changes relevant to phenotype.

## Additional file

Additional file 1: **Figure S1.** The “Y observed” vs. “Y predicted” diagram for the PLS-DA model of (A, B) serum and (D, E) tissue sample. The area under the curve (AUC) was 1 for both serum (C) and tissue (F) models for all the comparisons. (“C”: control group, “I”: rats with indomethacin-induced gastric ulcer, “I + AW”: ulcerated rats treated with AW extract). (TIF 427 kb)

## Data Availability

Data of this study are included in the article and the primary data can be provided from the corresponding author.

## Abbreviations

Alb: Albumin
AUC: Area under curve
AW: *Achillea wilhelmsii C. Koch*
C: Control
CMC: Carboxy-methyl cellulose
Csta: Cystatin-A
DTT: Dithiothreitol
Fabp5: Fatty acid binding protein/epidermal
Hspb1: Heat shock protein beta-1
I: Indomethacin
Lgals: Galectin-7
NSAIDs: Non-steroidal anti-inflammatory drugs
PCA: Principal component analysis
PEG2: Prostaglandin E2
RMSE: Root mean square error
ROC: Operating characteristic curve
Tagln: Transgelin
