# Supplementary figures and images for: Therapeutic effects of hydro-alcoholic extract of Achillea wilhelmsii C. Koch on indomethacin-induced gastric ulcer in rats: a proteomic and metabolomic approach
Source: BMC Complement Altern Med. 2019 Aug 7;19:205. doi: 10.1186/s12906-019-2623-4 (PMC6686504; doi:10.1186/s12906-019-2623-4)

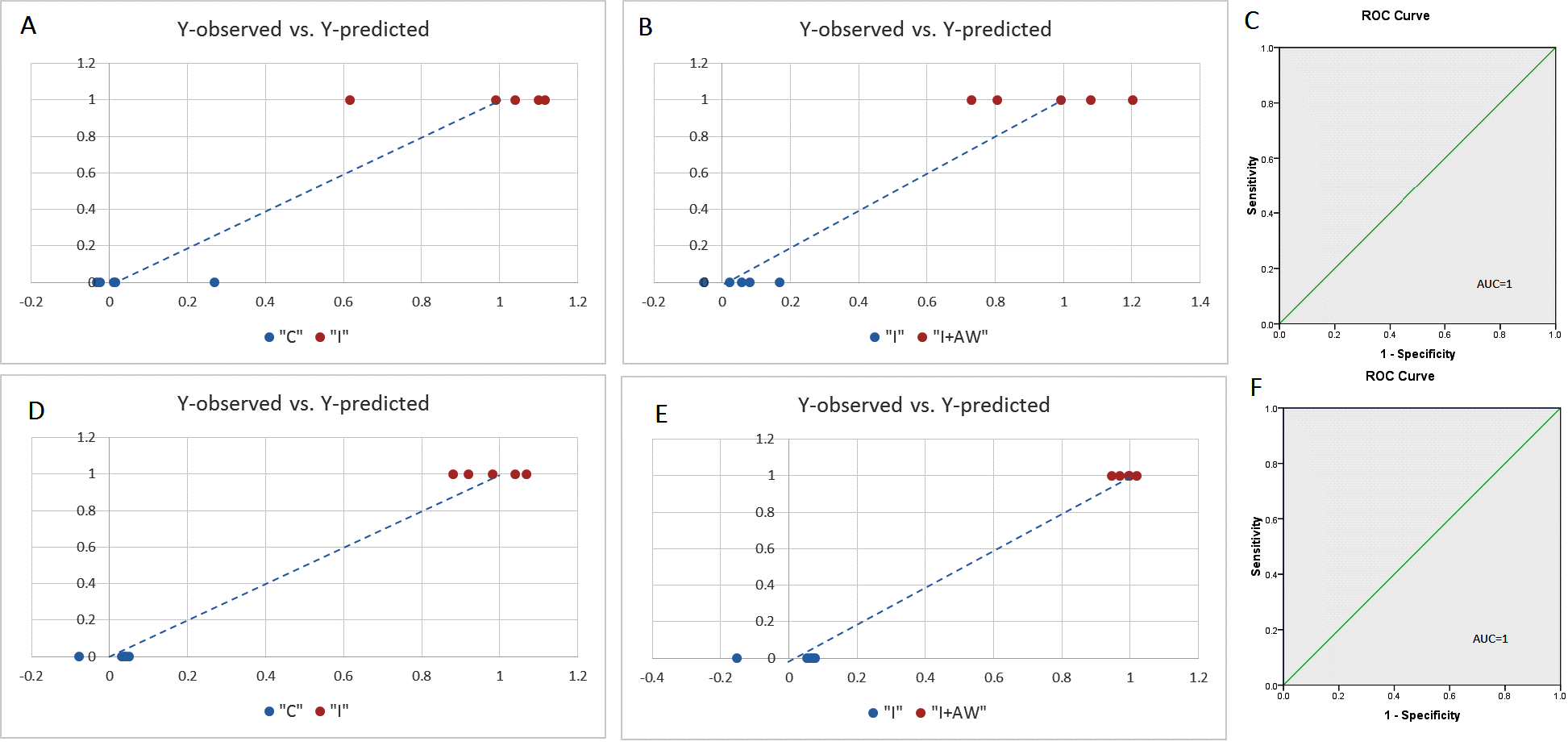

Supplement: Supplementary file 1 — Figure S1. The “Y observed” vs. “Y predicted” diagram for the PLS-DA model of (A, B) serum and (D, E) tissue sample. The area under the curve (AUC) was 1 for both serum (C) and tissue (F) models for all the comparisons. (“C”: control group, “I”: rats with indomethacin-induced gastric ulcer, “I + AW”: ulcerated rats treated with AW extract). (TIF 427 kb) [file 12906_2019_2623_MOESM1_ESM.tif]
